# Seasonality of cholera from 1974 to 2005: a review of global patterns

**DOI:** 10.1186/1476-072X-7-31

**Published:** 2008-06-20

**Authors:** Michael Emch, Caryl Feldacker, M Sirajul Islam, Mohammad Ali

**Affiliations:** 1 Department of Geography, University of North Carolina-Chapel Hill, USA; 2 Carolina Population Center, University of North Carolina-Chapel Hill, USA; 3 Department of Health Behavior and Health Education, University of North Carolina-Chapel Hill, USA; 4 ICDDR, B: Centre for Health and Population Research, Bangladesh; 5 International Vaccine Institute, Korea

## Abstract

**Background:**

The seasonality of cholera is described in various study areas throughout the world. However, no study examines how temporal cycles of the disease vary around the world or reviews its hypothesized causes. This paper reviews the literature on the seasonality of cholera and describes its temporal cycles by compiling and analyzing 32 years of global cholera data. This paper also provides a detailed literature review on regional patterns and environmental and climatic drivers of cholera patterns.

**Data, Methods, and Results:**

Cholera data are compiled from 1974 to 2005 from the World Health Organization Weekly Epidemiological Reports, a database that includes all reported cholera cases in 140 countries. The data are analyzed to measure whether season, latitude, and their interaction are significantly associated with the country-level number of outbreaks in each of the 12 preceding months using separate negative binomial regression models for northern, southern, and combined hemispheres. Likelihood ratios tests are used to determine the model of best fit. The results suggest that cholera outbreaks demonstrate seasonal patterns in higher absolute latitudes, but closer to the equator, cholera outbreaks do not follow a clear seasonal pattern.

**Conclusion:**

The findings suggest that environmental and climatic factors partially control the temporal variability of cholera. These results also indirectly contribute to the growing debate about the effects of climate change and global warming. As climate change threatens to increase global temperature, resulting rises in sea levels and temperatures may influence the temporal fluctuations of cholera, potentially increasing the frequency and duration of cholera outbreaks.

## Background

### Introduction

This paper systematically describes the seasonality of cholera in different parts of the world and comprehensively reviews scientific literature that investigates why seasonal patterns exist. The causative agent of cholera, *Vibrio cholerae *(*V. cholerae *hereafter), is a water-borne bacterium that is a natural inhabitant of brackish aquatic environments. Cholera is an acute infection caused by the colonization and multiplication of *V. cholerae *O1 or O139 within the human small intestine. People contract cholera when they ingest an infective dose of *V. cholerae *from contaminated water or food. Many developing countries still endure frequent outbreaks due to the lack of basic sanitation services and clean water.

Previous studies describe the temporal variation of cholera in localized study areas [1-18]. Many investigators postulate that the temporal variation of the disease is due to environmental and climatic factors that affect the seasonal patterns of infection [1,9-13,19-25]. Despite this growing interest in the causes and patterns of cholera, no global review of cholera's seasonal variations or the hypothesized mechanisms causing these patterns has been published. This is partially due to the fact that there are only a small number of comprehensive cholera surveillance systems in affected areas. Thus, few empirical studies rigorously examine the seasonality of cholera, leaving our understanding of variations of cholera outbreaks in different regions of the world incomplete. To fill this gap, this paper explores the seasonal cycles of cholera around the world using 32 years of data compiled by the World Health Organization (WHO) in the Weekly Epidemiological Record [26]. The analysis includes all reported cholera cases by date and country between 1974 and 2005. While these data include only reported cases, and select countries such as Bangladesh do not regularly report cholera to the WHO, this global dataset is the only systematic source of its kind and offers a unique opportunity to analyze global and regional patterns of cholera.

### Literature Review

#### Evidence of seasonality of cholera

Cholera infections vary greatly in frequency, severity, and duration, and the endemicity of cholera in different parts of the world is dynamic. Cholera is firmly endemic in some areas such as the South Asian countries of Bangladesh and India where cholera infections occur every year. In contrast, other regions such as parts of South America and Africa have historically had only sporadic epidemics. Yet, even in areas of persistent, endemic cholera, the magnitude of yearly epidemics varies dramatically from year to year.

In Bangladesh, several studies describe a regular seasonal cycle for cholera outbreaks, including specific studies on the different strains: classical, [5] El Tor, [27] and O139 [9]. While the symptoms of classical, El Tor, and O139 cholera are similar, some differences in their seasonal cycles are reported. Merson et al.[28], Glass et al. [29], and Samadi et al. [5] each describe a dominant seasonal cycle for classical cholera that is later than then peak of the newer strain, *V. cholerae *El Tor; El Tor is most incident from September to November, just after the monsoon. Several additional studies describe this pattern of two annual El Tor cholera peaks: a smaller spring outbreak in April before the monsoons followed by a larger fall outbreak from September to December after the monsoon [10,11,23,30-41]. Emch and Ali [42] describe a similar seasonal pattern for another new strain, *V. cholerae *0139, when it first appeared in Bangladesh in 1993. These patterns are evident in other areas of the region as well. In Pakistan, classical cholera typically increases from November to January and from April to May [43,44] while in Kolkata, India, seasonal patterns of cholera cases peak in April, May, and June[35,37].

Few surveillance systems collect detailed cholera incidence data outside of South Asia; therefore, information on the seasonality of cholera outside of this region is more limited. In South America, seasonal peaks are reported in summer months, January to February [10,32,33,37,41] as well as with the rise in waters following the rainy season in Amazonia, Brazil [45]. Major cholera outbreaks are recorded in eastern African nations including Djibouti, Kenya, Mozambique, Somalia, Uganda and Tanzania where the majority of outbreaks occur following rainfall and/or floods [46]. These cholera peaks also coincide with the summer rains. Mhalu et al. [6] reports that from 1979 to 1983 there were two cholera peaks in Dar es Salaam, Tanzania from October to December and again from March to May, both coinciding with periods of increased rainfall. In rural southern Tanzania, the peak of the cholera epidemic is slightly later, in June and July [47]. Bateman [48] reported the 2002 cholera high season for the northern parts of southern Africa occurred from the last week of January to mid-March. This period of heightened cholera is slightly longer in Mozambique and occurs during the hot, rainy months from December to May [49,50].

#### Evidence of environmental drivers

*V. cholerae *inhabit seas, estuaries, brackish waters, rivers, and ponds of coastal areas of the tropical world [51,52]. They flourish in the dense organic matter, algae, and zooplankton of the Ganges delta and similar ecosystems. It is probable that primary transmission to humans is enabled by micro- and macro-level environmental factors such as temperature, salinity, nutrient concentrations, the number of available attachment sites (plankton), shellfish consumption, and contact with water [53]. These factors are, in turn, influenced by larger-scale climate variability [10].

Although the seasonality of cholera is apparent, until recently the reservoirs that enable the survival and multiplication of *V. cholerae *during interepidemic periods were unknown. During epidemics, *V. cholerae *are isolated from patients as well as from surface water; however, during inter-epidemic seasons, *V. cholerae *largely cannot be cultured from the environment [22,23,54-58]. Recent studies provide possible explanations of how seasonality and endemicity of cholera are maintained [59,60]. The seasonal cycles of cholera appear to be closely associated with changes in flora and fauna populations in the coastal environment [56].

Biotic and abiotic parameters of water appear to provide temporary and long-term biotic reservoirs for cholera. These relationships are examined in Asia, primarily in Bangladesh. The overall seasonal fluctuation in *V. cholerae *may be due to the seasonal variation in physical and nutritional aquatic parameters [17] including conditions in both oceans and the brackish ponds and canals of rural Bangladesh [61-63]. *V. cholerae *survival is also dependent on abiotic characteristics including alkalinity, salinity, and iron [20,24,54,55,59,64-67] that influence the expression of virulence genes and regulate the cholera toxin that causes watery diarrhea [10,68]. Salinity may also partially explain the seasonal variation of cholera [66,67,69]. *V. cholerae *may be unable to persist in winter with colder water temperatures, but aquatic reservoirs with salinities of 0.25 to 3.0% and temperatures consistently above 5°C may maintain cholera in endemic areas [70].

Microbiological studies in Bangladesh reveal additional details about the cycle of dormancy and activity of *V. cholerae *within environmental reservoirs [1,23,56,71,72]. *V. cholerae *secrete an enzyme called mucinase that degrades mucin in the environment [73]. As mucin is present in plant cell walls, early studies suggest the association between *V. cholerae *and aquatic plants [21,74]. Additional studies on *V. cholerae *O1 confirm this hypothesis and find that various aquatic plants (e.g., water hyacinth, marine algae, duck weed, cyanobacteria) can act as a temporary reservoir [22,24,55] while blue green algae (*Anabaena spp*.) can act as a long-term reservoir [1,21]. During interepidemic periods, toxigenic *V. cholerae *residing within aquatic plants do not lose their pathogenic properties [22,23,75] but become largely nonculturable [22,54,76-78]. It is likely that during cholera epidemics the bio-physicochemical parameters of estuaries are ideal for the multiplication and transmission of *V. cholerae*, and as a result, these water sources will be heavily contaminated with *V. cholerae *[74,79].

Changes in the aquatic environment also affect the seasonality of cholera because the growth of the phytoplankton and aquatic plants provide food for zooplankton. Zooplankton and cyanobacteria are also potential reservoirs of *V. cholerae *[56,64,80,81]. Viable but noncultureable *V. cholerae *are detectable in association with both cyanobacteria and zooplankton from the aquatic environment in Bangladesh [56,72,77,82,83]. *V. cholerae *O1 secrete an enzyme called chitinase, which is associated with chitinous fauna, mainly zooplankton [84,85]. Zooplankton may provide attachment sites for *V. cholerae *to multiply and serve as a vector to transmit an infective dose to humans [10,61,86]. Others concur and demonstrate that the post-monsoon epidemic is associated with a heavy bloom of phytoplankton and zooplankton [59,63,86-88]. After an initial lag period, *V. cholerae *proliferate and are subsequently transmitted to humans [36]. A recent hypothesis about environmental controls of cholera suggests that vibriophages regulate epidemics. Faruque et al [17] show that the inverse correlation between vibriophages and susceptible *V. cholerae *strains implies that epidemics are more likely to begin in periods of low phage concentration (i.e. after floods and the monsoon season).

#### Macro-level factors

Macro-level associations and patterns between climate and cholera can be indirectly measured illustrating additional mechanisms that influence seasonality. Pascual et al.[15] posits that the temporal variability of cholera is associated with three interrelated climate variables that include upper troposphere humidity, cloud cover, and top-of-atmosphere absorbed solar radiation. Lipp et al.[10] adds that climate variability (i.e., climate change, El Nino-Southern Oscillation [ENSO], North Atlantic Oscillation), seasonal effects (i.e., sunlight, temperature, precipitation, monsoons), and human dimensions (i.e., socioeconomics, demographics, and sanitation) are also key drivers of outbreaks. Similarly, ENSO raises water temperature, bringing about increased zooplankton blooms [10] that may influence longer cycles in cholera periodicity, including a 4-year fluctuation pattern [89].

Using satellite imagery, Lobitz et al [90] find a correlation between sea surface temperature, sea surface height, and cholera in the Bay of Bengal in Dhaka, Bangladesh from 1992 to 1995. They suggest that sea surface height increases human-*vibrio *contact by transporting the bacteria into inland waters through the tidal intrusion of plankton [90]. Climate can affect the temperature of the sea surface as well as local ponds and rivers, possibly increasing the incidence of cholera through the faster growth rate of the pathogen in aquatic environments [16,36]. The heating of surface water may lead to an increase in phytoplankton blooms which feed zooplankton, encouraging the subsequent multiplication of commensal copepods that house *V. cholerae *[10,83].

Periodic climatic and temperature cycles such as ENSO have an effect on inter-annual cholera variability. To understand these seasonal and inter-annual patterns, analysis at larger spatial scales is necessary [14]. The periodic effects of ENSO, including the warming of the Pacific Ocean off the coast of South America, correlate with a 2001 outbreak of cholera in Peru after a century long hiatus [2,91,92]. Epstein [93] notes that warm El Niño events are linked to cholera outbreaks in Bangladesh and also to the emergence of new harmful algal blooms throughout Asia. Koelle et al [2] found that cholera outbreaks in Bangladesh between 1966 and 2002 demonstrate a nine to fourteen month lag between Indian Ocean sea surface temperatures and atmospheric temperature changes and subsequent cholera outbreaks (examples include '87-88 El Niño, '88-89 La Niña, and the '97-98 El Niño). Rodo et al [89] further support the role of ENSO by identifying the change in variability of cholera between the past (1893–1940) and present (1980–2001) as the result of a more prominent role of climate forcing by current ENSO effects. Overall, inter-annual cycles are important indicators of large scale patterns in cholera seasonality and of anomalous seasonal outbreaks [14,89]. Greater emphasis on seasonal anomalies in conjunction with yearly averages would aid understanding of these inter-annual outbreak patterns [13].

#### Secondary infections and human transmission

The seasonality of cholera outbreaks may also be explained by secondary transmission. Factors affecting secondary transmission mandate the extent to which the disease is present, i.e. whether it will reach epidemic proportions [7]. Several studies find that the severity of secondary transmission is associated with local environmental variables, predominantly water sources for household consumption. People who use contaminated surface water for drinking, cooking and bathing are more likely to contract cholera than those who do not [94,95]. In epidemiological work, studies identify an inverse relationship between diarrhea and access to tube well water [96]; positive associations with canal water compared with river or pond water [57]; and higher cholera incidence rates in villages that are not adjacent to rivers [29]. Other studies find associations between cholera and flood control [34,97]. While these studies show that the local environment (e.g., water and sanitation) facilitates secondary transmission of cholera, it cannot fully explain why the disease has such predictable temporal patterns.

## Materials and methods

### Data

This paper describes the seasonality of cholera throughout the world during a 32-year period using WHO Weekly Epidemiological Record of all reported cases by date from 1974 to 2005 [26]. The Weekly Record compiles data from the World Health Organization's global cholera surveillance system. Each Weekly Record provides information about outbreaks in each country and including the number of reported cases, deaths, and the cholera serotype, when available. The Weekly Record does not consistently include within-country details such as the specific region of the outbreak. A project database is compiled that includes the number of cholera outbreaks for 140 countries for each of the 12 calendar months. An outbreak is defined as at least one reported cholera case in any calendar month. Multiple outbreaks during the same calendar month of the same year are treated as one outbreak. Outbreaks are used instead of disease rates due to the differences in surveillance systems. Some countries report cases more accurately than others, and there are always more cholera cases than those that are reported.

### Methods

Dichotomous season variables are developed using WHO cholera case data. In the northern hemisphere, season one is December through May and season two is June through November; the seasons are reversed for the southern hemisphere. The latitude for each country is recorded using the centroid (i.e., the geographic center) of each country, assigning one latitude value to each. To ensure adequate model fit, the latitude variable is considered to be an absolute value, representing the distance to the equator. In addition, an ordinal variable is created to categorize individual countries within a geographic range represented by latitude values (0°–4°, 5°–9°, 10°–19°, 20°–29°, 30°–39°, 40°–49°, 50°–59°, and 60°+).

The outcome variable is the country-level number of outbreaks in each of the 12 preceding months. Because this variable represents a count over a fixed time period, a Poisson loglinear regression is initially considered using the model, ln(μ) = β_0 _+ β_1_[abs(latitude)] + β_2_(season). However, the χ^2^/df (Pearson's chi-squared divided by the degrees of freedom) far exceeds one (approximately 4.3), providing evidence of overdispersion. Therefore, the Poisson model would be ineffective since the mean does not equal the variance. Instead, we use the negative binomial. The negative binomial distribution is robust to overdispersion and allows the variance to equal μ + k* μ^2^, where k is the overdispersion factor and μ the mean. The negative binomial output in SAS provides an ancillary parameter, α, which is an estimate of the degree of overdispersion. If overdispersion is present, α will be greater than one.

For the combined hemisphere model, the model of best fit is the saturated model with season, latitude, and interaction of season and latitude. To reach this conclusion, and test whether certain parameters in a model are zero, likelihood-ratio tests are conducted. To test the significance of the interaction term, the saturated model is compared to a model without the interaction term; the test result has an associated p-value of < .0001, providing evidence that the interaction term is non-zero. An overall test to see whether the saturated model, including season and latitude with interaction, fits better than the simple intercept model yields a p-value of < .0001 showing at least one, non-zero parameter. For the northern hemisphere model, the model of best fit also contains the saturated model of season and latitude with interaction. Likelihood-ratio tests are used to examine the intercept versus the saturated model as well as the model with just season and latitude versus the saturated model. These tests provide evidence that the saturated model is the model of best fit. Lastly, for the southern hemisphere model, the model of best fit includes only latitude. Because the seasons are reversed, the estimates confidence interval for latitude would be the negative of the value listed. The p-value would remain the same. A likelihood-ratio test is conducted to determine if the interaction term is zero: the resulting chi-square value of 0.36 with 1 degree of freedom and p-value > 0.25 provides evidence that the parameter is zero and should be dropped from the model. To determine if further simplification is possible, a likelihood-ratio test between the model with season and latitude is compared to the model with only latitude. The resulting test (p-value ~ 0.1) provides evidence that season can be dropped from the model. Lastly, a likelihood-ratio test between the intercept model and a model with only latitude has an associated p-value of < .0001. Therefore, we conclude that the model of best fit for our data is one containing only the parameter latitude.

To map these results by country, we create a cholera seasonality index. To create this index, the total number of June to November outbreaks are subtracted from the total number of December to May outbreaks for each country, yielding a positive number, a negative number, or a zero. Subsequently, that number is divided by the total number of outbreaks (December to May outbreaks + June to November outbreaks), yielding a positive or negative number, ranging from -1 to 1, for each country. The absolute value of this number shows seasonality, regardless of hemisphere. These values are broken into 8 categories of increasing seasonality. A number close to 1 indicates strong seasonality. A number close to 0 indicates no cholera seasonality.

## Results

**Figure 1:**
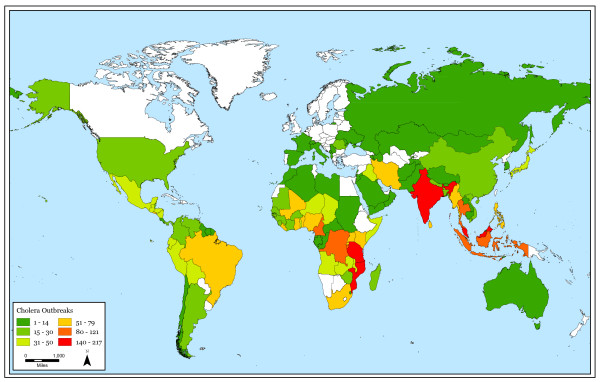
Total number of cholera outbreaks, 1974–2005.

**Figure 2:**
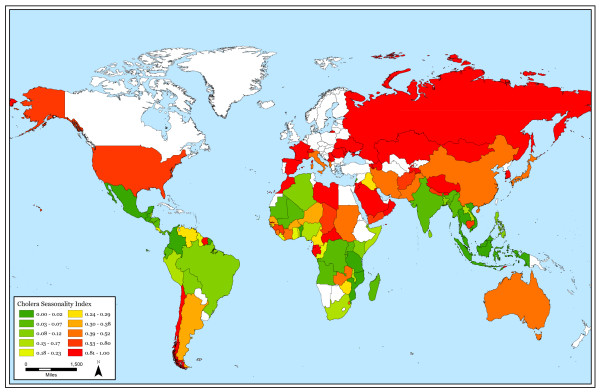
Cholera outbreak seasonality, 1974–2005.

**Figure 3:**
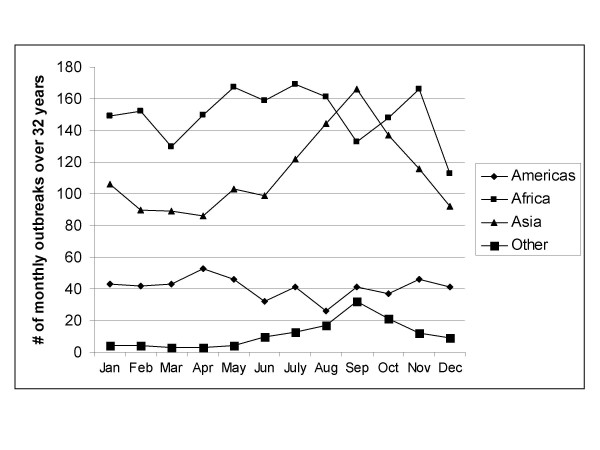
Monthly cholera outbreaks by region.

**Figure 4:**
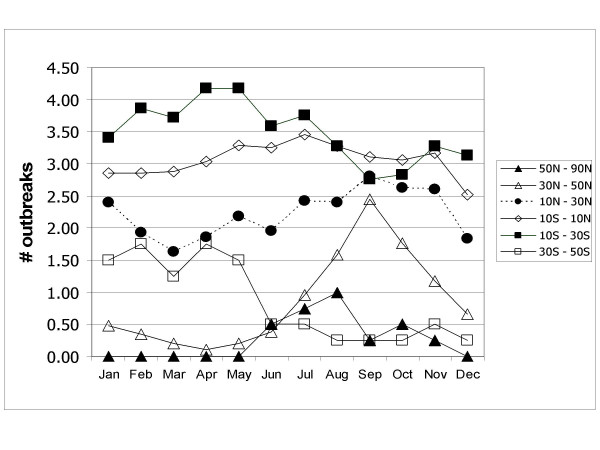
Average # of monthly cholera outbreaks by latitude.

**Figure 5:**
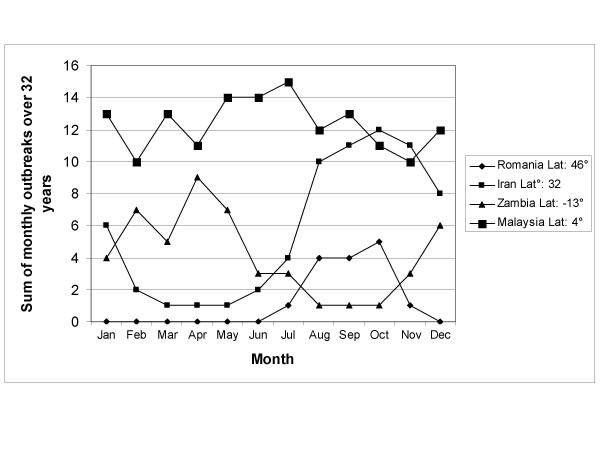
Distinct seasonal cholera patterns in four countries.

**Figure 6:**
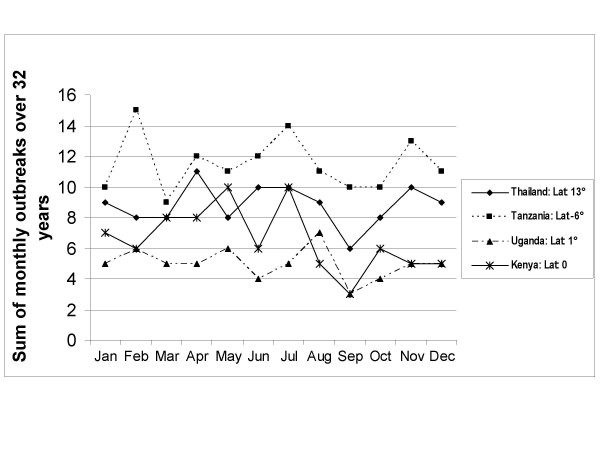
Cholera patterns in four countries within 15 degrees of the equator.

Seasonal patterns persist in higher absolute latitudes but cholera outbreaks do not follow a clear seasonal pattern near the equator. Also, cholera outbreaks occur more often closer to the equator than at higher latitudes. Figure 1 shows the total number of cholera outbreaks by country for the 32-year study period. Although there are exceptions, this figure illustrates that there are more outbreaks in countries near the equator in comparison to countries in higher absolute latitudes. Figure 2 displays the cholera seasonality index to show the strength of cholera seasonality in each country. The graphic shows that stronger seasonal patterns (in red) appear more prevalent in countries further from the equator. Figure 3 shows the total number of outbreaks per region over 32 years. Although annual peaks are evident, it is difficult to determine distinct seasonal patterns in cholera outbreaks across regions. However, grouping countries by latitude range, rather than region, makes these seasonal peaks more obvious. Figure 4 groups countries within absolute latitude ranges showing the average number of outbreaks per month over the 32-year period. Figure 5 illustrates seasonal cholera patterns in four countries, Romania, Iran, Zambia, and Malaysia. These four countries are purposefully selected because they represent different latitude ranges, including one from the southern hemisphere, and demonstrate clear seasonal patterns. The 10 to 30 north range has a similar pattern to the 30 to 50 north range, but the 30 to 50 range shows a dramatic seasonal peak. The peaks become more obvious moving from the 10 to 30 south range to the 30 to 50 south range as well. The seasonal cycles are especially clear when comparing the 30 to 50 north and south latitude ranges: they have the exact opposite seasonal cycles. Among lower latitude ranges, seasonal cycles are either non-existent or show few clear patterns. Figure 6 illustrates the total number of cholera outbreaks by month for four countries within 15 degrees of the equator, Thailand, Tanzania, Uganda, and Kenya. These countries are purposefully selected to represent equatorial countries with sizeable cholera cases that do not show dramatic seasonal variation.

The results from the statistical models confirm these visible seasonal patterns. Table 1 shows that for the combined hemispheres statistical model, both latitude and latitude*season interaction are significantly associated with the number of cholera outbreaks in the preceding 12 months. For season one (December-May), the multiplicative effect on the number of expected outbreaks for each 1-unit increase in latitude is 0.94. There is a 6% decrease in the expected number of outbreaks for each 1-unit increase in latitude in season 1. This conclusion is reached by using the following equation, and adding the common exponentiated factors: e^-.12*1^e^-.09 [latitude]^e^.03*1*latitude ^= e^-.12^e^-.06 [latitude]^. For example, for a latitude of 20°, the number of expected outbreaks for season two (June-November) is e^0.47 ^= 1.61 times the expected number for season one. In other words, for latitude of 20°, there is a 61% increase in the number of expected outbreaks for season two compared to season one according to the equation: e^-.12[season]^e^-.09*20^e^.03*season*20 ^= e^-1.76 ^e^0.47*season^.

**Table 1:** Influences on Cholera Seasonality: Combined Hemisphere Model

Variables	Model-Season	Model-Latitude	Combined Model	Combined Model with Interaction
Season	0.29*		0.34*	-0.12
Latitude		0.04*	-.04*	-0.09*
Season*Latitude				0.03*
				
Over-dispersion	1.777	1.482	1.444	1.418
Deviance	1714	1713	1706	1693
Pearson Chi-Square	1633	1892	1966	2069
Log-Likelihood	676.8	767	783.1	798.1
N	1678	1678	1678	1678

*p < .01

For the model including only countries in the northern hemisphere, both latitude and seasonality are important (Table 2). For season one, the multiplicative effect on the number of expected outbreaks for each 1-unit increase in latitude is e^-.058 ^= 0.94; therefore, there is a 6% decrease in the expected number of outbreaks for each 1-unit increase in latitude. For latitude of 20°, the number of expected outbreaks for season two is e^0.47 ^= 1.61 times the expected number for season one. For 20° latitude, there is a 61% increase in the number of expected outbreaks for season two compared to season one

**Table 2:** Influences on Cholera Seasonality: Northern Hemisphere Model

Variables	Model-Season	Model-Latitude	Combined Model	Combined Model with Interaction
Season	0.35*		0.47*	-0.15
Latitude		-0.04*	-0.04*	-0.09*
Season*Latitude				0.03*
				
Over-dispersion	1.921	1.636	1.582	1.551
Deviance	1267	1266	1259	1248
Pearson Chi-Square	1362	1563	1648	1760
Log-Likelihood	196.1	254.4	269.7	281.9
N	1282	1282	1282	1282

*p < .01

For the southern hemisphere only model, latitude is the most important variable (Table 3). Season is not significant in the model, and the season*latitude interaction is only marginally significant at the 0.1% level. The multiplicative effect on the number of expected outbreaks for each 1-unit increase in latitude is e^-.036 ^= 0.96; therefore, there is a 4% decrease in the expected number of outbreaks for each 1-unit increase in latitude in the southern hemisphere.

**Table 3:** Influences on Cholera Seasonality: Southern Hemisphere Model

Variables	Model-Season	Model-Latitude	Combined Model	Combined Model with Interaction
Season	0.18		0.22**	-0.03
Latitude		-0.04*	-0.04*	-0.07*
Season*Latitude				0.02**
				
Over-dispersion	1.229	1.096	1.083	1.074
Deviance	441.1	440.7	440.3	439.7
Pearson Chi-Square	329.1	327.3	334.8	337.0
Log-Likelihood	506.8	521.8	523.8	524.0
N	394	394	394	394

*p < .01, **p < .10

## Discussion

The results suggest the existence of seasonal patterns of cholera outbreaks over the last 32 years and demonstrate that these patterns differ by latitude. The patterns are evident in visual representation and confirmed by statistical analysis. In higher latitudes in both hemispheres, cholera outbreaks exhibit seasonal patterns while seasonal patterns do not persist near the equator. Cholera outbreaks are also more common near the equator. These findings suggest that larger climactic factors may be at play in the appearance of *V. cholerae*. These macro-level climatic factors are certainly related to a combination of complex local-level parameters that are described in the literature review section of this paper. This paper reviews many of the postulated reasons for temporal fluctuations of cholera, including both seasonal cycles and interannual variability. Although it is still difficult to determine whether the models linking cholera incidence to environmental parameters are valid outside of particular study areas, this paper reflects global trends. The body of evidence suggests that cholera is tied to environmental and temporal parameters ranging from local to global scales. Our study complements previous research by showing that cholera fluctuations are linked to latitude, and this confirms our expectation of seasonal cholera patterns over time. These findings are also consistent with a disease that is linked to climate, and it appears that there is an association between cholera and both micro- and macro-level environmental parameters. Ongoing research investigates the relationship between cholera and both environmental and climate variables in several local sites around the world where there is detailed cholera and population data [60].

There are several limitations to this study. Primarily, the WHO cholera surveillance database is incomplete. Not all cholera outbreaks are reported to the WHO, and some countries have better reporting systems than others. Other countries, such as Bangladesh, do not regularly report to the WHO, so a complete record of all global cholera outbreaks does not exist. Additionally, the WHO Weekly Record does not always provide details on the specific location or serotype of the outbreak, so it is not possible to examine within-country variation in cholera seasonality or differences in seasonal patterns by cholera serotype. However, the dataset is adequate to show general patterns around the world despite missing data. There are other very important local environment and climate parameters (rainfall, temperature, regional differences, etc.) that require further consideration in future studies. Despite the limitations, this study is a unique opportunity to gain insight into cholera patterns around the globe. The authors believe that the trends presented in this analysis would hold if additional data were included.

## Conclusion

These results indirectly contribute to the growing debate about the effects of climate change and global warming. As climate change threatens to increase global temperature, resulting rises in sea levels and temperatures may influence the temporal fluctuations of cholera, potentially increasing the frequency and duration of cholera outbreaks. As found by this study, countries near the equator, which generally have higher and more constant temperatures, have a greater and more constant level of cholera outbreaks. Countries further from the equator in higher absolute latitudes typically have seasonal cholera outbreaks, mostly in the warmer months, and they have lower overall outbreak levels. However, climate change may influence the strength, duration, or appearance of these annual seasonal patterns. The potential alterations in seasonal cholera outbreaks may leave some countries unprepared for outbreaks outside of previously-recorded seasonal patterns. Greater numbers of cholera outbreaks and more unpredictability may increase both morbidity and mortality.

## Competing interests

The authors declare that they have no competing interests.

## Authors' contributions

ME conceived of the study and supervised all aspects of its implementation, CF and ME wrote the paper and MSI helped write the background section, MA helped with the study design and statistical analysis.
